# SubcloneSeeker: a computational framework for reconstructing tumor clone structure for cancer variant interpretation and prioritization

**DOI:** 10.1186/s13059-014-0443-x

**Published:** 2014-08-26

**Authors:** Yi Qiao, Aaron R Quinlan, Amir A Jazaeri, Roeland GW Verhaak, David A Wheeler, Gabor T Marth

**Affiliations:** Department of Biology, Boston College, 140 Commonwealth Ave, Chestnut Hill, MA 02135 USA; Department of Public Health Sciences and Center for Public Health Genomics, University of Virginia Health System, Charlottesville, VA 22908 USA; Division of Gynecologic Oncology, Department of Obstetrics & Gynecology, University of Virginia Health System, Charlottesville, VA 22908 USA; Department of Bioinformatics and Computational Biology, The University of Texas MD Anderson Cancer Center, 1515 Holcombe Blvd, Huston, TX 77030 USA; Human Genome Sequencing Center, Department of Molecular and Human Genetics, Baylor College of Medicine, MS BCM226, One Baylor Plaza, Huston, TX 77030 USA; Department of Human Genetics and USTAR Center for Genetic Discovery, Eccles Institute of Human Genetics, University of Utah School of Medicine, 15 North 2030 East, Salt Lake City, UT 84112 USA

## Abstract

Many tumors are composed of genetically divergent cell subpopulations. We report SubcloneSeeker, a package capable of exhaustive identification of subclone structures and evolutionary histories with bulk somatic variant allele frequency measurements from tumor biopsies. We present a statistical framework to elucidate whether specific sets of mutations are present within the same subclones, and the order in which they occur. We demonstrate how subclone reconstruction provides crucial information about tumorigenesis and relapse mechanisms; guides functional study by variant prioritization, and has the potential as a rational basis for informed therapeutic strategies for the patient. SubcloneSeeker is available at: https://github.com/yiq/SubcloneSeeker.

**Electronic supplementary material:**

The online version of this article (doi:10.1186/s13059-014-0443-x) contains supplementary material, which is available to authorized users.

## Background

Identifying the few genetic changes that drive chemo-resistance or metastasis from hundreds or thousands of somatic variants found in whole-exome or whole-genome sequencing [1,2] of matched tumor-normal patient tissue samples is a daunting task. Current variant prioritization approaches examine predicted variant impact in candidate genes, or deploy pathway analysis to narrow down the long list of candidate mutations to a manageable number [3]. Here we report an alternative approach to variant prioritization, exploiting the patterns of genetic heterogeneity often observed in diverse types of cancers.

The presence of such genetically divergent subpopulations of cells within a single tumor mass has been reported in various tumor types [4-23]. In contrast to normal tissue, in which the same germline mutation is present in every cell, a somatic mutation may be present in some, but not all, cancer cells within a tumor biopsy as a result of rapid mitotic growth and continuous selection. With multiple groups of somatic mutations present at different cellular frequencies, the tumor mass consists of distinct populations of cells, or tumor subclones, with each subclone harboring a specific subset of the mutations. The ability to delineate each such clonal subpopulation, determine its frequency within the tumor mass, and to infer the evolutionary relationships among subclones allows one to determine the order in which the mutation events occurred, and permits the identification of those mutations that are most likely to play a part in tumorigenesis, drug response, relapse, and metastasis.

Earlier studies have attempted to reconstruct subclonal structure with many different methods typically tailored to their specific study designs. These methods fall into distinct classes including: (1) cell genotype profiling using *in situ* hybridization [4,5]; (2) identifying distinct allele frequency (AF) modals by clustering, followed by subclone structure reconstruction via visual inspection of the data and manual reasoning [6-13]; (3) phylogenic reconstruction based on single-cell PCR or sequencing-based profiling [14-20]; and (4) phylogenetic reconstruction using biopsies gathered from multiple metastases [21-23]. While each method adequately addressed the dataset in which it was applied, neither provided a sufficiently general framework for subclone reconstruction from somatic variation data. The work we are presenting is focused on automating the ‘reasoning’ step that starts with somatic variants from matched tumor/normal tissues of a single cancer patient, as well as additional tissues (for example, relapse, metastasis) if available, and ends in the enumeration of possibly multiple subclone structures consistent with the input data, and additional derived information that may be useful for variant prioritization or guiding treatment. The main difficulty of subclone reconstruction is the fact that the AFs measured in a large population of tumor cells, as is the case in ‘bulk tissue’ tumor sequencing or microarray genotyping experiments, do not retain the underlying linkage information that exists between individual somatic events, that is, whether or not two or more mutation events are present within the same cell. Unfortunately, given *n* mutation events, there are in total *n!* possible subclone structures, and often a large number of these can account for the AF measurements equally well. This makes it very difficult or impossible to unambiguously reconstruct subclone evolution from per-locus AF observations. To address these challenges, computational methods have been recently developed for tumor tissue purity estimation (that is, partitioning tumor cell populations into a mixture of normal and tumor subpopulations), using microarray [24-26] or sequencing data [27-29]. Even more recently, multiple algorithms to reconstruct clonal structures were developed. These algorithms either exploit specific biological assumptions [30] to choose between many mathematically equivalent structures; or by using statistical sampling procedures [31] to explore the solution space of all possible subclone structures. Both of these methods require high-precision AF measurements of one specific variant type: somatic single nucleotide variants or SNVs, and (presumably because of the computational complexity involved) only produce results for up to a few input sites (see Supplemental Result 1 in Additional file 1, and the datasets used in Additional files 2 and 3). Other approaches utilize maximum likelihood mixture decomposition on CNV data input [32]; jointly estimate subclone genotypes with only SNV [33] or with both CNV and SNV data [34], but without requiring that the subclones they infer fit within a consistent phylogeny; or model the possibly multi-furcating tumor phylogeny with a bifurcating tree, without the ability to consider multiple tumors from a single patient (such as primary / relapse pairs) [35]. There have also been several methods developed in the context of transcriptome data, which are summarized in a recent review article [36].

Here we present a more general approach based on a strategy that is able to accept many types of somatic variation data (for example, SNVs, or copy number variations from sequencing or microarray datasets, Figure S2 and S3 in Additional file 1. Refer to Additional file 4 for sample datasets and scripts) as input. Out method enumerates all possible subclone structures that are consistent with the bulk AF measurements from the input data. It is capable of reducing this solution space significantly, often to a single, unique solution when data from multiple tumor biopsies such as primary and relapse from the same patient are available. In the event that more than a single alternative subclone structure still remains after such trimming, it is often possible to derive high-confidence linkage information between subsets of loci based on the consensus of all remaining structures. In such cases, we focus not on efforts to disambiguate mathematically equivalent solutions, but rather on using the complete set after our pruning procedure in a statistical framework to determine, for example, the probability that two given mutations are present within the same subclone (mutation co-localization), or that a given mutation pre-dates another one (mutation order). Such co-localization information may reveal, for example, that two distinct mutations that each sensitizes the cancer cells to specific drugs are, in fact, present on a single subclone. Given the high incidence and therapeutic challenges posed by chemoresistant tumors, knowledge of mutation co-localization may allow for more accurate and potentially more efficacious targeted therapeutics aimed at countering or preventing chemoresistance. Moreover, if such a novel mutation in a chemo-resistant tumor is present in every cell of the relapse sample, it may be a top candidate in the search for a mutation driving chemo-resistance.

## Results and discussion

### Our computational procedure for subclone structure analysis

**Figure 1:**
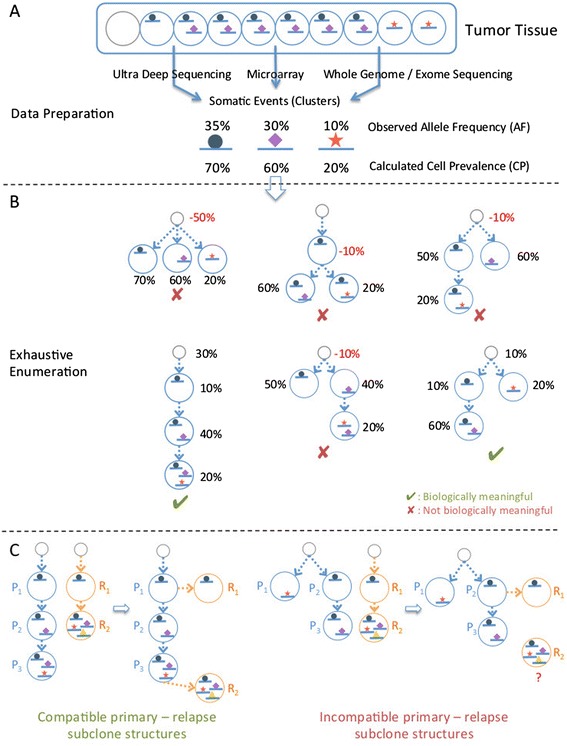
**SubcloneSeeker method overview. (A)** Data preparation: genomic variation data (SNVs, CNVs, and so on) are converted into the corresponding cell prevalence (CP) values, and clustered into distinct groups. **(B)** Structure enumeration: based on the identified CP clusters, all possible subclone structures, represented as branching tree structures where one subclone is derived from its ‘predecessor’ by the addition of a mutation (or cluster of mutations), are visited. During the visit, each subclone on the tree structure is assigned a subclone frequency (SF) value so that the implied total CP values for mutations are in agreement with the input CP values. Those structures with negative SF values are removed from the solution set. **(C)** Solution trimming: the aim of this procedure is to merge the subclone structures from the relapse tumor (orange circles) those from the primary tumor (blue circles) from the same patient. Left panel: example showing a compatible pair of relapse/primary structures. Right panel: example showing a pair of incompatible relapse / primary subclone structures. A subclone in the relapse, R_2_, cannot be positioned anywhere within the primary subclone structure because it contains mutations found in separate primary subclones (P_1_ and P_3_.), and therefore cannot be derived from either one or the other.

Here we briefly describe the main characteristics of the algorithm to investigate the relationships among somatic events from unlinked, bulk allele frequency measurements at somatic mutation sites (Figure 1, section ‘Method’).

#### A unified framework for subclone structure reconstruction that incorporates all types of genomic variants

We define a subclone as a collection of cells in the tumor sample that harbor the same set of genomic variants, including SNVs, structural variations (SV), copy number variations (CNV), loss of heterozygosity (LOH), and so on. The only requirement for a data type to be included in the analysis is the ability to derive the fraction of the cells within the tumor sample in which this mutation is present, a quantity that has also been referred to as ‘cell prevalence’ (CP) [37]. In a simplified example, a heterozygous SNV in a copy number neutral region with an AF of 30% would correspond to a CP of 60% (Figure 1A). The estimation of CP is no trivial task, especially for SNVs falling into regions of CNV, because the same measured allele frequency results in different CP value depending on the absolute copy number state in the region. A number of tools have been developed to facilitate CP calculation, including ASCAT [25] and ABSOLUTE [26], which estimates the absolute copy number states of CNV regions, and PyClone [37], which estimates CP from SNV allele frequency while taking into account copy number. Our method requires as input CP measurements, regardless whether these measurements represent SNVs, CNVs, or some other type of genetic variation, allowing it to consider each such variant type, or any combination of variant types from a given sample. We note that, as a preprocessing step, our method clusters together variants with the same (or similar) CP values to minimize measurement uncertainties, and assumes *a priori* that all variants in each such cluster are co-localized in the same cells. The input to our downstream methods is an ordered list of CP values, corresponding to those clusters.

#### Subclone structure reconstruction

**Figure 2:**
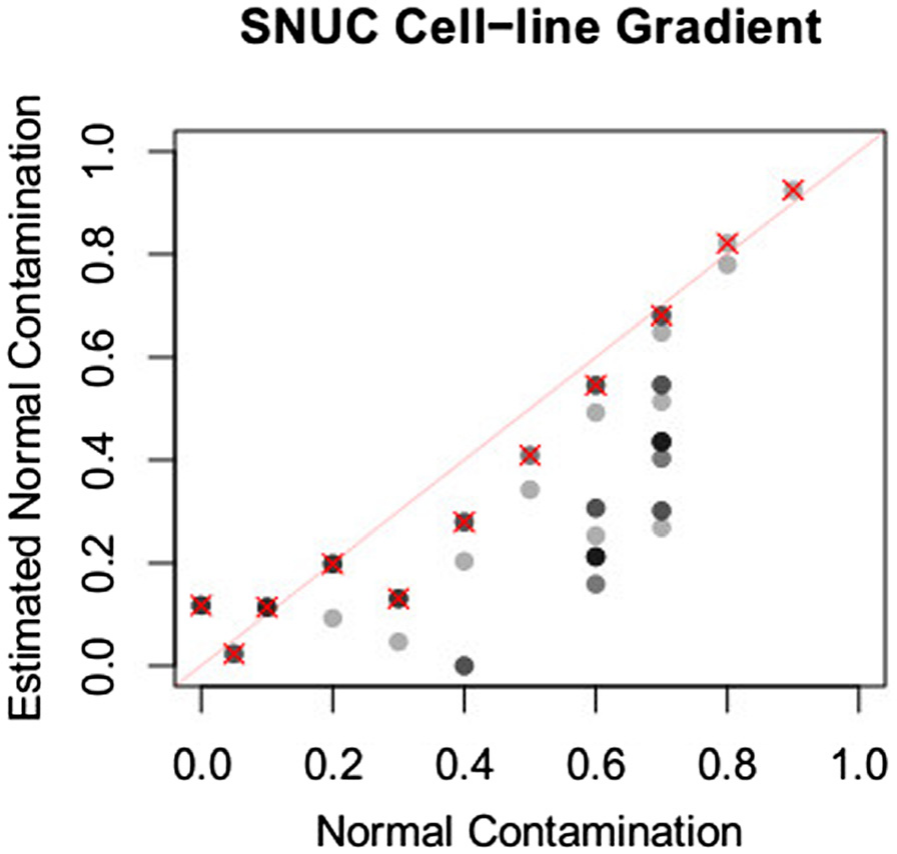
**Normal cell content estimated by subclone reconstruction in a controlled mixing experiment.** Dataset is generated by mixing sequencing reads from a SNUC cell-line and matched normal tissue. Data points corresponding to the subclone structure representing linear mutation accumulation are shown with a red cross.

Given *n* somatic events (clusters), each with an associated, distinct CP value, we enumerate all possible ‘evolutionary trees’ where mutation events occurring along the tree branches give rise to new subclones in a successive fashion (Figure 1B). For *n* mutations (clusters), this procedure results in *n!* distinct subclone structures assuming that: (1) cells in a tumor mass are derived from normal tissue cells or existing tumor cells through mitosis, in which recombination is unlikely to occur; and (2) the same mutation event does not spontaneously occur in two different subclones, nor does a mutation get lost from a subclone. Each subclone structure contains exactly *n* distinct subclones with associated subclone frequencies (SF), plus a ‘null’ subclone without any mutation, representing the normal tissue component within the tumor sample (and its SF the ‘normal tissue contamination’). SF is assigned to each subclone so that all subclones within a given structure, when put together, give rise to the same mutation (clusters) CP list as the input. In order to satisfy this condition, our procedure may need to assign negative SF values to one or more subclones; such subclone structures are not biologically plausible, and are removed from further consideration. As demonstrated later (Figure 2), only a small fraction of the structures are biologically plausible (we term these ‘viable subclone structures’).

#### Trimming the space of viable subclone structures

Often there are more than one viable subclone structures in the resulting solution set, corresponding to multiple alternative subclone evolutions. However, if additional ‘linkage’ data are available, further trimming is usually possible. Such linkage information may be either directly observed, such as in the case of spectral karyotype images [38-40], single cell colony assays, or single cell sequencing; or indirectly inferred from, for example, primary and relapse tumor from the same patient. Because typically, the relapse tumor is derived from the primary tumor, they share mutations originating from common ancestor subclones, and through such shared evolutionary history the primary and relapse subclones can be merged into one unified subclone structure (or multiple alternative unified subclone structures). Figure 1C shows examples of two compatible primary/relapse structures (left) as well as two incompatible ones (right). In the latter example, the relapse subclone R_2_ contains two mutations that are found in different branches on the primary tree (P_1_ and P_3_), violating the assumptions above. Any structure in the primary that has no compatible structure in the relapse, or *vice versa*, is discarded from consideration, reducing the solution space.

#### Mutation localization prediction

**Figure 3:**
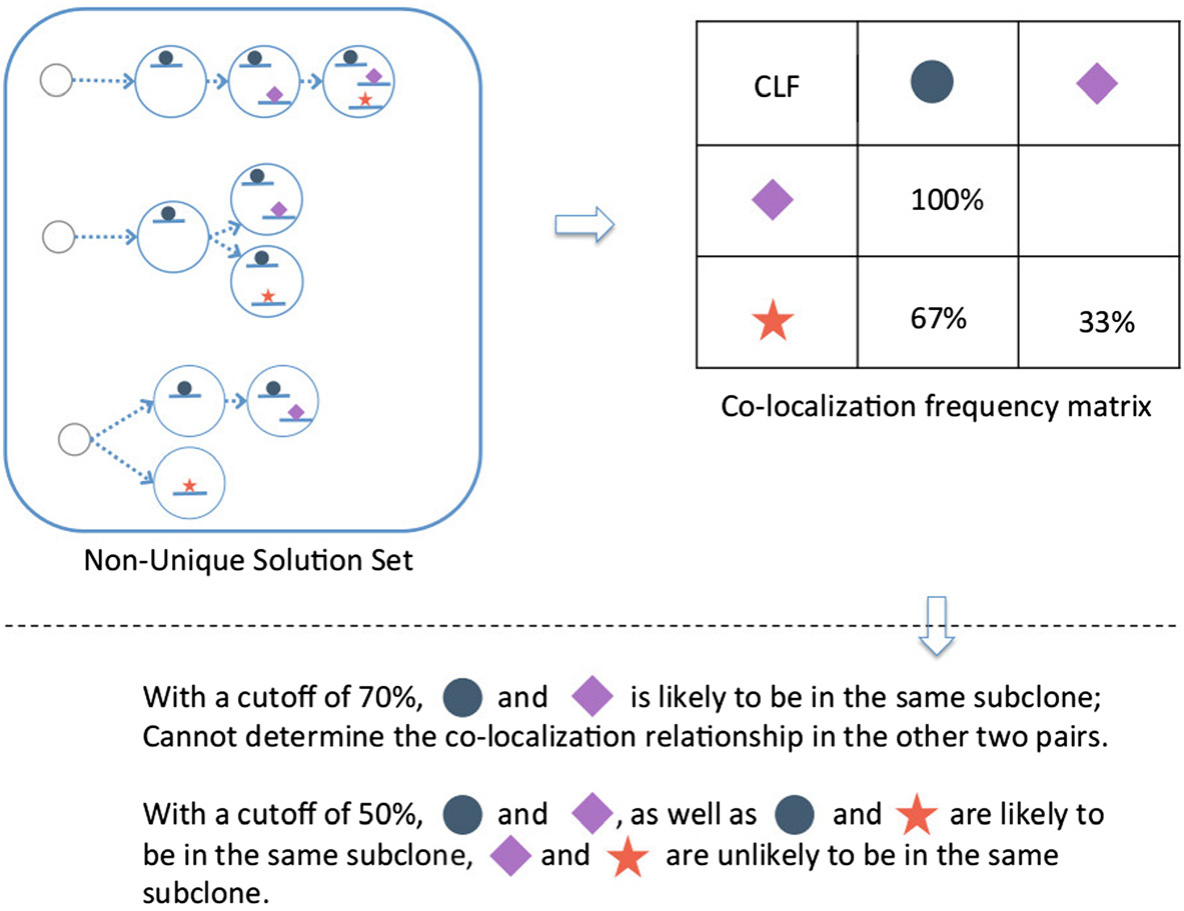
**Predicting mutation co-localization.** In cases where there are multiple viable subclone structures, we count the fraction of all structures within which two mutation events are co-localized. This fraction is the probability that the two events are present in the same subclone. One can also make a ‘co-localization call’ by declaring that two events are co-localized, if this probability is above a pre-defined threshold.

Useful knowledge can be derived even in cases where there are multiple alternative subclone structures. Although one cannot determine the precise subclone evolution with certainty in such cases, the collection of all possible solutions can be used to predict whether or not two mutations are present in the same cell, that is, whether or not they are co-localized within the same subclone. This prediction is based on the fraction of all viable subclone structures in which two mutations (or more generally, a given set of mutations) are present in at least one subclone. Such information could potentially be important in, for example, designing personalized chemotherapy treatment plans. Given n clusters, there are in total nC2 (n choose 2) unique, unordered cluster pairs, each of which is assigned a status of either ‘co-localized’, ‘not co-localized’, or ‘ambiguous’ (Figure 3, ‘Methods’). Furthermore, for two mutation events that are localized in the same subclone, the timing of the mutations can be easily determined: the event with the higher CP value appeared earlier, and the event with the lower CP value emerged later.

### The SubcloneSeeker software

SubcloneSeeker is implemented in C++, and its source code available under MIT license. The package provides a complete set of APIs and data structures to represent subclone and genomic mutation data types, along with well documented source code and examples, so that anyone can easily extend on the core functions we provided to incorporate domain-specific knowledge, such as placing different prior probabilities over tree structures.

### Our subclone structure reconstruction method always includes the correct structure among the solution set it reports

We generated simulated tumor samples (Supplemental Method 1 in Additional file 1) comprising 3, 4, …, 8 mutation events with distinct CP values. For each of these ‘tumor samples’, we produced a random subclone structure serving as a ‘true’ structure. We repeated this procedure 1,000 times. In every case, SubcloneSeeker was able to reproduce the ‘true’ subclone structure as one of the solutions in the complete solution set of viable subclone structures. This ‘sanity check’ was necessary to ensure that our software worked appropriately for simulated datasets.

### The number of biologically plausible subclone structures is low

**Figure 4:**
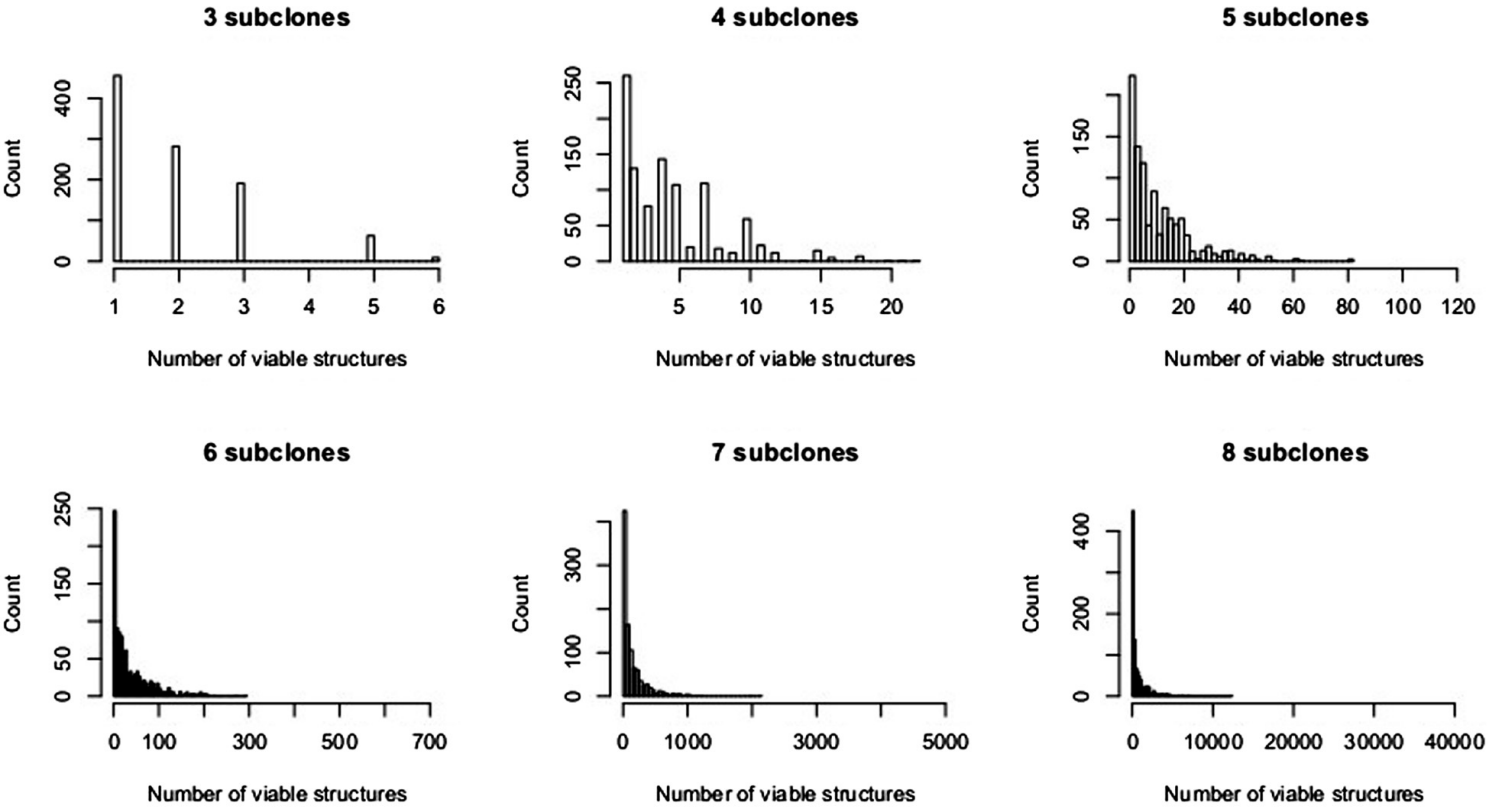
**Number of biologically meaningful structures histogram based on simulation.** Each plot is based on a set of 1,000 randomly generated subclone structures. The maximum value on the x axis of each plot represent the theoretical upper limit on the number of solutions, *n!*, given *n* CP values. The distributions are heavily compressed towards the left, suggesting that the actual number of biologically meaningful structures is usually small.

We also found that the number of viable subclone structures is very low compared to the number of all possible structures. As Figure 4 illustrates, the expected number of viable subclone structures is far less than the theoretical upper-limit (*n!* for *n* distinct CP values).

### Our normal cell component estimation procedure is accurate

As described above, our subclone structure reconstruction method provides, for each structure, each subclone present together with a subclone fraction, that is, the fraction of that subclone within the tumor biopsy. The structure includes a subclone without any of the mutations: this is the normal cell component of the tumor biopsy, and its fraction is the normal cell fraction. We investigated the accuracy with which our method estimates the normal cell fraction in experimental data. We applied our method to a dataset created by mixing 10%, 20%, …, 90%, 95%, and 100% sequencing reads from a SNUC (Sinonasal Undifferentiated Carcinoma) cell line sample [41], with reads sequenced from paired normal tissue (Figure 2). In this dataset, the non-branching, stepwise mutation accumulation model (red-cross), a parsimonious solution that always exists (section ‘Method’), produced very accurate estimate for normal cell content among all alternative structures (R^2^ = 0.9705395 to the line y = x).

### Our algorithmic procedure for subclone structure comparison improves on interpretation in previously published data

In a recent study, Ding *et al.* [7] investigated clonal evolution in eight acute myeloid leukemia (AML) patients. To ensure easy comparison with the published results, we started with the somatic mutation clusters and AF values provided in the study (Table S5c and Table S10 in Ding *et al.* Additional file 1), rather than re-computing them ourselves. With two exceptions, SubcloneSeeker produced the same subclone structures, and with one exception, came to the same biological conclusions (Table S1 in Additional file 1).

**Figure 5:**
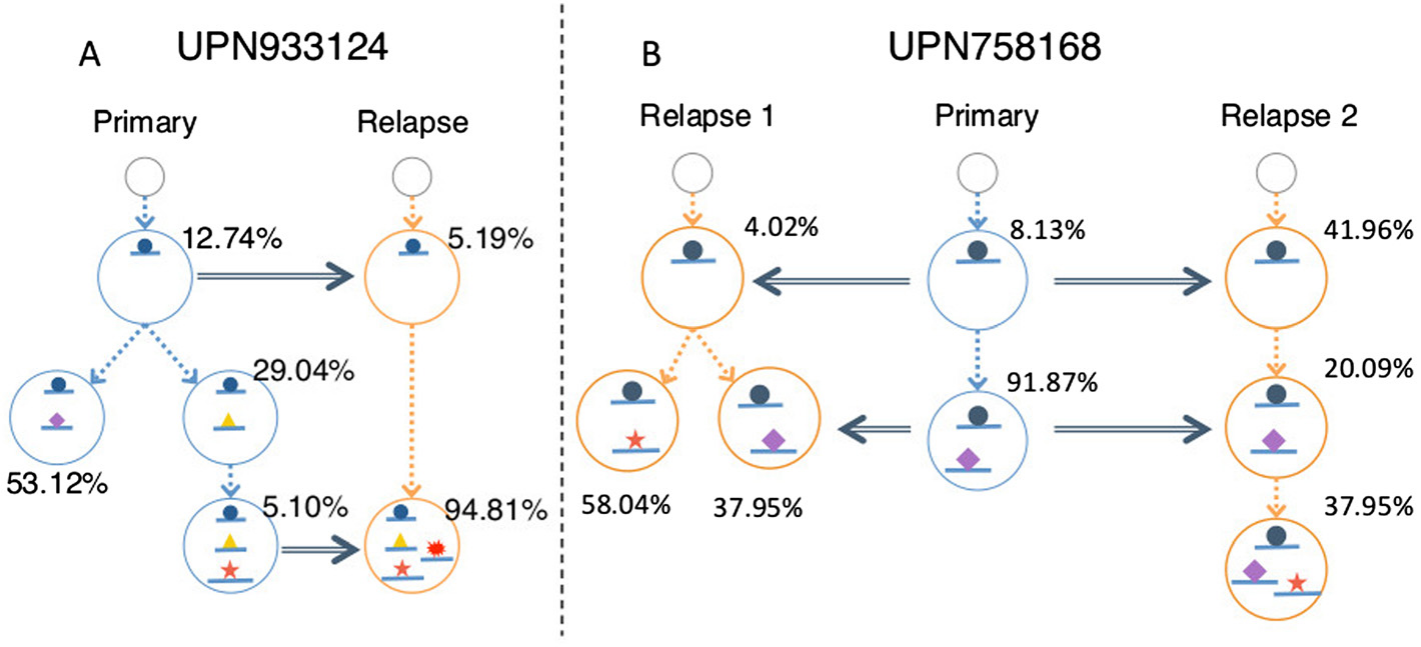
**Our re-analysis of published primary/relapse AML dataset in Ding** ***et al.*** Primary, relapse, and merged subclone structures for two patients, reconstructed with SubcloneSeeker. **(A)** SubcloneSeeker analysis found six alternative primary subclone structures for patient UPN933124. Only one is compatible with the relapse subclone structure, and the pair is in agreement with the original study. **(B)** Each of the two viable merged primary/relapse subclone structures for patient UPN75816 suggests that the two primary subclones made it to the relapse tumor, and further expanded.

In the case of patient UPN933124, the primary sample contained two low frequency clusters, which resulted in a total of six different viable subclonal structures, including the one reported in the original study. However, only one of these was compatible with the sole viable subclone structure in the relapse, and the resulting single primary/relapse subclone structure was in agreement with the model presented in the original paper (Figure 5A). In the case of patient UPN758168, the relapse sample yielded two possible structures, both of which were compatible with the primary structure. However, the tumor expansion model suggested by either of these structures disagrees with the expansion model described in the original paper as ‘a minor clone carrying the vast majority of the primary tumor mutations survived and expanded at relapse’. Our subclone structures (Figure 5B) suggest, in contrast, that both primary subclones survived in the relapse. The difference between the two relapse models is which primary subclone expanded with extra mutations.

### Analysis of TCGA primary-relapse ovarian tumor samples reveals two distinct patterns for tumor recursion in the dataset

**Figure 6:**
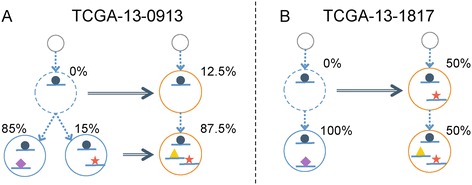
**Two relapse patterns in a TCGA primary-relapse ovarian tumor dataset. (A)** TCGA-13-0913 exemplifies a recursion pattern in which the relapse subclones are originated from multiple subclones in the primary, suggesting inadequate primary treatment. **(B)** TCGA-13-1817 signifies a different pattern in which one subclone in the primary acquired new mutations, became resistant to primary chemotherapy, and gave rise to the entire relapse tumor mass.

We applied SubcloneSeeker to a dataset of 17 ovarian cancer primary / relapse patients included in the TCGA ovarian serious carcinoma cohort [42]. We observed two distinct relapse patterns in this dataset (manuscript in preparation). The first pattern, exemplified by TCGA-13-0913 (Figure 6A) and observed in five of the 17 patients, is one where multiple subclones found in the relapse are already present in the primary tumor, suggesting that chemotherapy against the primary tumor was inadequate. The second pattern, exemplified by patient TCGA-13-1817 (Figure 6B) and observed in eight of the 17 patients, is one where relapse tumor subclones descended from a single, rare, and therefore unobserved primary subclone, and acquired new mutations that might now confer resistance to the chemotherapy used against the primary tumor.

### Analysis of whole-exome sequencing data from chemo-resistant *versus* primary ovarian tumors demonstrates that our subclone structure analysis can be used to prioritize somatic mutations for further follow-up

**Figure 7:**
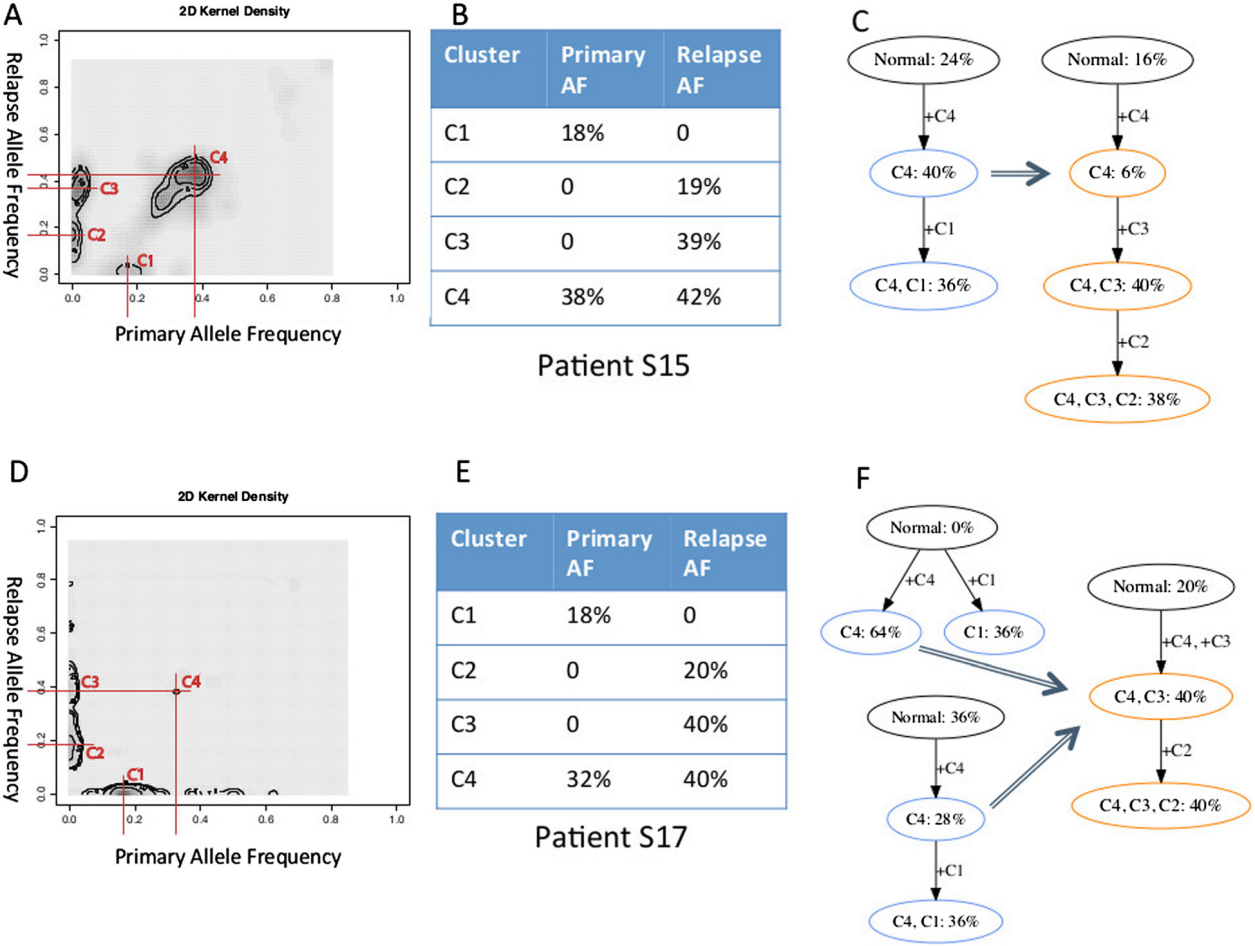
**Analysis of whole-exome sequencing data on patient S15 and S17 from chemo-resistant relapse vs. primary ovarian cancer dataset. (A)** Clustering of somatic mutations in patient S15. **(B)** Mutation clusters and CP values in S15 primary and relapse. **(C)** Uniquely identified, compatible S15 primary and relapse subclone evolution tree. **(D)** Clustering of S17 somatic mutations in patient S17. **(E)** Mutation clusters and CP values in S17 primary and relapse. **(F)** Two viable structures for S17 primary, and a sole structure for S17 relapse.

We are investigating how high-grade serous ovarian cancers become chemoresistant by applying SubcloneSeeker to whole exome sequencing datasets on normal, primary tumor and chemoresistant relapse tumor tissue samples from the same patient. Figure 7 shows our analysis workflow for prioritizing mutations observed in patients ‘S15’ and ‘S17’. Somatic mutations were first clustered in the ‘Primary AF – Relapse AF’ space to identify discrete modals, corresponding to distinct subclones (Figure 7A, B, D, E). The allele frequencies of these clusters were then converted to cell prevalence values, and subjected to subclone structure reconstruction. Certain ‘abnormal’ SNVs with AF values between 0.5 and 1 are likely to be in CNV regions. Due to the nature of exome sequencing, we do not have reliable CNV estimations to perform accurate correction. Out of necessity, we ignore the variants with greater than 50% AFs. In the case of ‘S15’, both the primary and the relapse sample yielded a unique structure; these are compatible with each other (Figure 7C). The mutations in mutation cluster ‘C4’ are early events in the primary, present in every cell of the relapse, and likely contain the driver mutation responsible for initial tumor expansion. On the other hand, in the relapse sample, the vast majority (93%) of tumor cells contain the mutations that make up cluster ‘C3’. This makes it likely that the mutation(s) conferring the chemoresistance phenotype are part of this cluster.

In the case of sample ‘S17’, the primary sample yielded two viable subclone structures, both compatible with the sole structure in the relapse (Figure 7F). Similarly to sample ‘S15’, mutation cluster ‘C4’ is likely to contain the initial driver mutation(s), and mutation cluster ‘C3’, which is present in all relapse subclones, is likely to contain the mutation leading to chemoresistance. In both samples, the use of subclone analysis resulted in information that one can use for variant prioritization, in order to narrow down the set of somatic events in the search for the causative mutation, both for initial tumor expansion, and for chemoresistance.

### Simulation studies demonstrate that our statistical framework is able to accurately predict whether two somatic mutations (or mutation clusters) are localized in a subclone together

**Figure 8:**
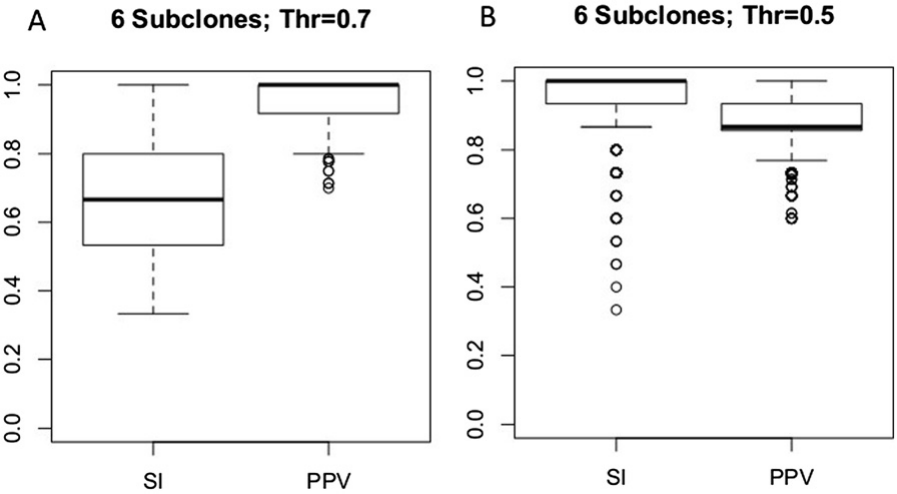
**Performance of mutation co-localization prediction on simulated data. (A)** Co-localization prediction statistics on simulated dataset with six subclones in each tumor sample, and a threshold of 0.7. SI - Combined Sensitivity; PPV - Combined positive predictive value; **(B)** Co-localization prediction statistics on simulated dataset with six subclones in each tumor sample, and a CLF threshold at 0.5.

To understand the behavior of our methods predicting co-localization of mutations within subclones, we simulated tumors with five, six, and seven subclones (in each case, 1,000 replicates), performed our subclone reconstruction procedure, and carried out mutation co-localization analysis (section ‘Method’). We used threshold values of 0.7 and 0.5 to call whether two mutations are co-localized, not co-localized, or that the results are ambiguous (see Figure 8 for six subclones, and Supplemental Figure S4 in Additional file 1 for the complete set). Importantly, at a call threshold of 0.7, our method calls co-localized and not co-localized pairs with approximately 70% sensitivity and nearly 100% positive predictive value (PPV, the fraction of correct calls in all the calls made). At a threshold of 0.5, sensitivity goes up to nearly 100%, while PPV drops to approximately 80%.

### Re-analysis of bulk *versus* single cell colony assay data demonstrates that we are able to accurately identify mutations that are present in the same subclone

**Figure 9:**
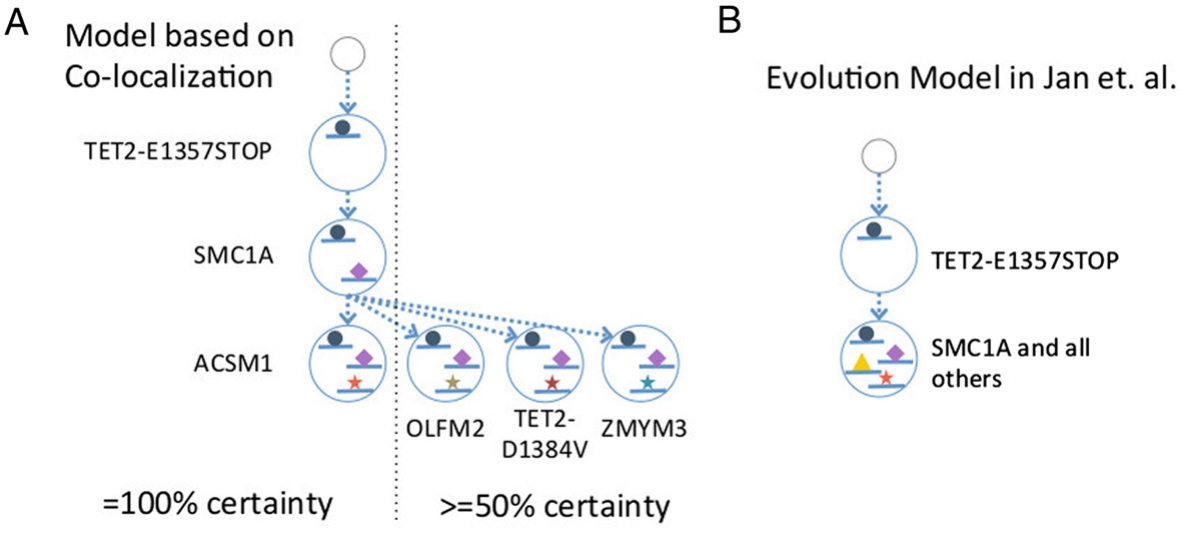
**Analysis results on patient SU048 HSC sample in Jan** ***et al.*** **(A)** Our model of subclone evolution constructed based on co-localization probabilities. Left: Consensus structure supported by all subclone structures. Right: Consensus structure supported by at least 50% of subclone structures. **(B)** Model of subclone evolution reported in Jan *et al.* constructed based on colony assay results.

In a recent study by Jan *et al*. [15], hematopoietic stem cells (HSC) from several AML patients were sequenced to >20,000 depth to measure somatic mutation allele frequencies at several targeted loci. In addition, colonies grown from single cells separated from the sample were subjected to allele-specific SNV TaqMan assay at the same SNV sites, resulting in direct observations of subclones within the tissue. We used the bulk AF values obtained from the sequencing data as input to our subclone reconstruction method, followed by our mutation co-localization prediction procedure. We then compared our co-localization predictions to the colony assay results. Among four patient samples for which colony assay data were available, SU030 and SU008 did not yield conclusive results because the allele frequencies at the tested sites were so low (well below 1%) that they were indistinguishable from measurement noise (see Table S2 in Additional file 1). SU070 yielded a unique subclone structure that is in agreement with the structure identified by colony assay (Figure S5 in Additional file 1). SU048 (Figure 9) produced a result set of 48 viable subclone structures. Every structure supports that TET2-E1375STOP is the earliest event, followed by SMC1A and ACSM1 (Figure 9A, Table S3 in Additional file 1). With a co-localization calling threshold of 0.5, TET2-D1384V, OLFM2, and ZMYM3 co-localize with TET2-E1375STOP and SMC1A, which is in agreement with the conclusion in the original analysis by Jan *et al.* that AML precursor HSC cells contain double mutations (presumably forming a compound heterozygote) in the TET2 gene. According to our analysis, TET2-E1375STOP and SMC1A are the two early events, and the two TET2 mutations are already present in the same, early subclone. This is biologically sensible given that TET2 is involved in DNA demethylation [43] and SMC1A in chromosome structure maintenance [44]. In addition, the depletion of TET2 in mouse model leads to HSC expansion [45,46], and the lack of SMC1A protein predicts poor survival in AML [47]. On the other hand, the relatively low co-localization probabilities among ACSM1, TET2-D1384V, OLFM2, and ZMYM3 suggest a branching structure for these mutations (Figure 9A), rather than linear mutation accumulation consistent with the colony assay for this patient (the colony assay found one cell in which all these mutations are present). This points out the relatively weak power of our method to resolve co-localization among mutations with very low allele frequencies, as such low frequency mutations can be placed with relative freedom on multiple branches of the evolutionary tree.

## Conclusion

In this paper we present a novel algorithm to elucidate tumor subclonal structure using as input cell prevalence values of individual, unlinked somatic mutations. In contrast to other methods that require SNV allele frequencies, our method is able to analyze many different types of genomic variant data, as long as allele frequency measurements can be converted into cell prevalence values. Because bulk mutation frequency measurements from fragmentary sequence data or per-site microarray measurements do not retain ‘linkage’ across such somatic variant sites, often there are many alternative subclone structures that can account for the input measurements. Our method exhaustively enumerates all such viable subclone structures. We were able to show that the number of solutions is usually much smaller than the theoretical upper limit. Often tumor tissues from multiple phases of tumor development (for example, primary and relapse biopsies) are available. In such cases, the number of subclone structures that are not only consistent with the respective input frequency data but also across, for example, the primary and the relapse is lower, further trimming the ‘solution space’, often to a single, unique structure. Using both simulations and experimental data, we have extensively characterized and validated our methods. We have illustrated with a number of datasets that this approach is often able to identify key patterns underlying tumor progression and relapse, including information to guide mutation prioritization.

In the case that the solution space cannot be further trimmed, we provide methods to derive useful knowledge, in terms of mutation cluster co-localization and timing. Our subclone structure enumeration procedure is exhaustive, and is free from the biases introduced by the choice of parameters or prior distributions often required for statistical sampling of the subclone structure solution space. We demonstrated that the co-localization and timing of mutations predicted from the HSC bulk targeted sequencing (Jan *et al.*) correlate well with their function, and can be used in a similar fashion to prioritize functional study.

Our analysis of previously published datasets and our own datasets suggests that SubcloneSeeker will be applicable for a number of clinical/biological problems. Using serous ovarian cancer as an illustrative example, we have demonstrated that chemoresistance and relapse in this disease is a clonally driven process, and that such clones can be either present in the primary tumor or ‘arise’ during progression or relapse. The patterns of temporal mutational order and cellular co-localization provide clinically relevant insight into the genomic basis for chemoresistance. In ovarian cancer, 80% of tumors are classified as chemosensitive while 20% of cancers progress during or recur shortly after platinum-based adjuvant chemotherapy. Unfortunately, there are no known genetic markers at present that can reliably predict inherent or acquired chemoresistance. This is likely the result of the complex and multifactorial biological basis for this phenotype. However, whereas one or a small number of them may not be informative, analysis of many resistant clones and identification of the corresponding mutational order and cellular co-localization may lead to a better understanding of chemoresistance, and form a rational basis for targeting the chemoresistant clones.

We envision similar utility for this type of analysis in advancing the current understanding of genomic alterations involved in the pre-malignant phases of cancer. Once again using ovarian cancer as a prototypical case, it has been established that TP53 mutations are ubiquitous and early events in serous ovarian carcinogenesis [42]. However, the prevalence of other recurrent somatic mutations is about 10% or less [42] suggesting that the additional requirements for transformation may be met through a combination of more diverse co-localized or temporally related somatic mutations (plus possible contributions from epigenetics and other molecular alterations,and so on). Thus genomic investigation of putative precursor lesion for serous carcinoma using approaches presented here is likely to identify subclonal hierarchies whose constituent mutations define cooperative classes on oncogenic event whose sum total results in malignant transformation.

## Method

The complete workflow of our method consists of the following steps (with details concerning each step below):

1. Depending on the type of input data, mutation events and their associated allele frequencies are called by detection methods
2. The allele frequencies of events are converted into cell prevalence, and then subjected to clustering. If more than one sample is available, the clustering will be done in a multidimensional space, in which the number of dimensions is equal to the number of samples.
3. The resulting somatic event groups (by CP) serves as the input to the SubcloneSeeker core algorithm. This will result in a set of solutions that are biologically meaningful, and mathematically consistent with the input.
4. Further trimming can be performed on the solution set, such as trying to merge multiple samples into a unified evolutionary tree.
5. Mutation (cluster) co-localization can be inferred from the solution set.

### Data preparation

Various types of raw data are processed, in data-type specific ways, into somatic events. Whole genome copy number measurement: this is done either by whole genome sequencing (WGS) or array comparative genomic hybridization (aCGH) measurement on paired tumor-normal samples from a cancer patient. In the case of WGS, read depth is measured within large genomic window (for example, 10 kb). For aCGH, hybridization probe intensities are measured, and often averaged across multiple probes. Relative copy number (RCN) measurement is obtained by normalizing tumor read depth or hybridization intensity first to the total amount of DNA per sample (for example, the total number of reads), followed by normalizing to the corresponding measurements in the normal sample. This normalization step eliminates germline events shared by the tumor and the normal tissue, and keeps somatic events. Whole genome LOH measurement: the whole genome B-allele frequency (BAF) measurement of the tumor sample is filtered to exclude those SNVs that are identified as homozygous in the paired-normal sample to generate somatic LOH event profile, and from it a mirrored BAF (mBAF) [48] profile is calculated by the following rule:

$$ mBAF=\left\{\begin{array}{c}\hfill BAF,\  if\ BAF\ge 0.5\hfill \\ {}\hfill 1-BAF,\  otherwise\hfill \end{array}\right. $$

Segmentation: the RCN derived from CNV or mBAF measurement is then subjected to segmentation algorithms, such as DNAcopy [49,50] or HMMSeg [51], to identify continuous regions with the same copy number of LOH state, and to delineate event boundaries of the corresponding events. SNV AF estimation: deep sequencing SNV data do not need to be segmented, however their allele frequencies needs to be accurately estimated, for example, using PyClone [37], which also performs CP estimation.

### Cell prevalence calculation

CP is defined as in what percent of all the cells being examined does one specific event exist. Different data types require different techniques to perform this calculation. Whole genome CNV events: for whole genome CNV events derived from either WGS RD or aCGH probe intensity data, it is important to have a good estimation or, better yet, direct measurement, on the ploidy (p) and purity (q). Various software packages already exist to estimate p and q, such as ASCAT [25], CNAnorm [52], and ABSOLUTE [26]. Moreover, an absolute copy number state (ACN) needs to be called for every CNV event from relative copy number state (RCN) that is usually in the form of log2(Tumor / Normal ratio). For examples shown in this paper, ACN is called with a Maximum Likelihood method (described below). In the case of whole genome sequencing, read depth were calculated by counting the number of reads falling in each of a 10 kbp non-overlapping window. Afterward, the read depth log2 ratio is obtained by:

$$ Log2 Ratio=lo{g}_2\left(\frac{R{D}_t}{R{D}_n}\right) $$

where *RD*_*t*_ and *RD*_*n*_ are the read depth of tumor and paired normal sample corrected for total DNA quantity, for any given window. Agilent aCGH microarray: same procedure was applied, only that read depth was substituted with probe intensity of each probe the array reported. However since the data file is already in log2 ratio form (with the samples comparing to a reference sample), the actual formula is slightly different:

$$ Log2 Ratio= Log2P{I}_t- Log2P{I}_n $$

where *Log*2*PI*_*t*_ and *Log*2*PI*_*n*_ are the log2 ratio of the probe intensity. This is because the probe intensity log2 ratio can be expressed in the form of:

$$ Log2P{I}_t=lo{g}_2\left(\frac{P{I}_t}{P{I}_{ref}}\right) $$

$$ Log2P{I}_n=lo{g}_2\left(\frac{P{I}_t}{P{I}_{ref}}\right) $$

thus

$$ \begin{array}{rcl} Log2 Ratio& =& lo{g}_2\left(\frac{P{I}_t}{P{I}_{ref}}\right)-lo{g}_2\left(\frac{P{I}_n}{P{I}_{ref}}\right)\\ {}\ & =& lo{g}_2\left(\frac{P{I}_t}{P{I}_n}\right)\end{array} $$

The ACN of each segment is assigned using maximum likelihood estimation, assuming equal probability for all possible subclone fractions. In the case of a diploid genome after correcting for purity, a deletion region with a segmental log2 ratio between 0 and −1 will be called as heterozygous deletion (unless significant high LOH is observed, in which case homozygous deletion is called), and a log2 ratio less than −1 will be called as homozygous deletion. Once ACN is estimated, CP can be calculated as

$$ \begin{array}{rcl}\because ACN\bullet CP+2\bullet \left(1-CP\right)& =& RCN\\ {}\therefore CP& =& \frac{RCN-2}{ACN-2}\end{array} $$

in which RCN is the relative, non-discrete copy number and ACN is the called, absolute copy number that only takes discrete integer value. LOH events: After segmentation of the mBAF data mentioned above, CP of each segment is calculated by the following equation

$$ CP = \frac{2u-1}{n\left(1-u\right)+\left(2u-1\right)} $$

in which *u* is the segmental mean and *n* is the ACN of the segment (which can be estimated by applying the CNV data processing technique described above to the LRR track of SNP6 microarray). SNVs: with accurate allele frequency estimation made available by ultra-deep sequencing and software advancements [37], CP can also be derived from SNVs along with allele specific copy number quantifications. For example, in diploid regions, *CP* = 2 ∙ *AF* for heterozygous SNVs, and *CP* = *AF* for homozygous SNVs.

### Clustering

Because the measurement of AF, and consequently CP, is potentially noisy, we attempt to mitigate its effect through clustering on CP to identify its modals. Examples shown in this paper are clustered with the kernel density function in R, with its bandwidth calculated by the Pilot Estimation of Derivatives [53]. Users can choose to substitute with more advanced techniques, such as MCLUST [54]. When multiple samples are available, it is important to perform clustering on multidimensional space, in which the dimension equals the number of samples, to identify separately inherited clusters.

### Subclone structure reconstruction

Let *E* = {*e*_*i*_}, *i* = 1.. *m* be the vector that contains all *m* observed somatic event clusters, *CP* = {*cp*_*i*_} be the vector contains the cell prevalence of each event cluster *e*_*i*_; *B*^*j*^ = {*b*_1_, …, *b*_*m*_}^*j*^, *j* = 1.. *n* be a row vector that the value ${b}_i^j$ indicates whether a specific subclone *j* has the somatic event belongs to event cluster *e*_*i*_; *f*^*j*^ ∈ (0, 1) be the fraction, out of the entire tumor, a specific subclone *j* takes up. A subclone structure with *n* subclones can be modeled as:

$$ {C}^j=\left\{E,{B}^j,{f}^j\right\},j=1..n $$

Thus the problem of subclone structure reconstruction can be stated as, for any given observation data in the form of {*E*, *CP*}, find *n* and *C* so that it would satisfy:

$$ \left[{f}^1,{f}^2,\dots, {f}^n\right]\kern0.5em \times \kern0.5em \left[\begin{array}{c}\hfill {B}^1\hfill \\ {}\hfill {B}^2\hfill \\ {}\hfill \vdots \hfill \\ {}\hfill {B}^n\hfill \end{array}\right]=CP $$

### Subclone evolution tree enumeration

Due to the unique biology of tumorigenesis, we make the following assumptions:

- Cells in a tumor mass are derived from germline cells or parental, existing tumor cells through mitosis, in which recombination is unlikely to occur.
- The same event (with respect to the boundary resolution) would not spontaneously occur in two subclones without a descendent relationship, nor would pre-existing events revert back to the normal state in a descendent subclone.

This means that if a late subclone *C*^*j*^ derived from an earlier subclone *C*^*k*^, $\forall i\kern0.75em \mathrm{that}\kern0.75em {b}_i^k=1,\kern0.75em \mathrm{we}\ \mathrm{have}\kern0.75em {b}_i^j=1$. Since no subclone can plausibly have a negative CP, this relationship means that the subclone in which a particular event with a higher CP cannot be the children of subclones containing events with a lower CP. Notice that there always exist one solution, in which the mutations with the highest CP appeared first, and subclones emerge in a linear mutation accumulation fashion, that is, subclone *j* contains all the mutations found in subclone (j-1), plus the *j*-th highest CP mutation (or mutation cluster). More generally, this property enables us to devise the following iterative algorithm to enumerate all possible structures.

The function ‘Evaluate(T)’ will, through a post-order tree traverse, try to assign a subclone frequency (*f*, or SF) value to each of the tree nodes so that at the end the subclone structure will result in the observed data (E). If the function visits a leaf node, it will assign the CP of the event clusters uniquely contained in the node; if the function visits an internal node, it will assign the CP of the event clusters uniquely contained in the node, minus the sum of the SF of all its descendent nodes. If it can do so without assigning any node a less-than-zero SF, that specific tree structure is recorded as a feasible solution.

This method will result in a tree-set, which contains all the possible ways to partition the observed event clusters into subclones, and the phylogeny between the subclones. One can choose to further trim the set by external or internal linkage information, or perform co-existence prediction.

### Cross-sample merging

As mentioned earlier, enumerating through the entire solution space usually results in ambiguous answers. Yet a very common clinical study scenario would contain data acquisition from primary/relapse/normal sample trios. Since the relapse tumor essentially represents a continuation of the evolution process from the primary, attempts can be made to further trim the solution space by trying to merge the nodes on the relapse tree onto the primary tree, while satisfying the following two conditions:

1. After merging, for any given non-leaf node, its children node must have all the mutations presented in the node itself (extra mutations are allowed).
2. No two branches shall have the same mutation simultaneously without sharing a common parent node who has that mutation.

These two conditions assure the fundamental assumptions concerning tumorigenesis aforementioned are met. Through this process, if a specific primary (or relapse) tree cannot be merged with any relapse (or primary) tree, that specific tree is then an invalid solution, and can be discarded.

### Co-localization prediction

When a solution set contains more than one solution, for any given pairs of somatic event clusters, a co-localization frequency matrix (CLF) can be calculated as:

$$ CLF = {\displaystyle \sum_{i=1}^{\#\  of\  solutions}}P{S}_i\bullet CL $$

in which *PS*_*i*_ is the probability that solution *i* is the correct solution, which in case no prior knowledge is available, can be calculated as

$$ P{S}_i=\frac{1}{\#\  of\  solutions} $$

*CL* is a binary variable that describes whether the given pair co-localize in solution *i*, which can either be 1, if in at least one subclone the event clusters co-localize, or 0, if in none of the subclones the event clusters co-localize. This framework allows us to estimate co-localization giving all structures equal possibility to be true, or weight towards, or against specific structures. (For example, one can reasonably argue that it is generally unlikely for a patient to develop two, separate tumor subclones without related by an common ancestor, thus placing a lower prior on those structures in which multiple subclones are derived directly from the normal tissue).

## Additional files

Additional file 1
**Supplemental Materials.**
Additional file 2
**The dataset of SNVs of TCGA-13-0913 primary and relapse samples.**
Additional file 3
**The dataset of CNVs of TCGA-13-0913 primary and relapse samples.**
Additional file 4
**Sample datasets and scripts for running SubcloneSeeker.**

## Abbreviations

aCGH: Array Comparative Genome Hybridization
ACN: Absolute copy number
AF: Allele frequency
AML: Acute myeloid leukemia
BAF: B-Allele frequency
CNV: Copy number variation
CP: Cell prevalence
HSC: Hematopoietic stem cell
LOH: Loss of heterozygosity
PPV: Positive predictive value
RCN: Relative copy number
SF: Subclone frequency
SI: Sensitivity
SNUC: Sinonasal undifferentiated carcinoma
SNV: Single nucleotide variant
SV: Structural variation
WGS: Whole genome sequencing
