# Supplementary material for: SubcloneSeeker: a computational framework for reconstructing tumor clone structure for cancer variant interpretation and prioritization
Source: Genome Biol. 2014 Aug 26;15(8):443. doi: 10.1186/s13059-014-0443-x (PMC4180956; doi:10.1186/s13059-014-0443-x)
Supplement: Additional file 1 — Supplemental Materials. [file 13059_2014_443_MOESM1_ESM.docx]

**SubcloneSeeker: a computational framework for reconstructing tumor clone structure for cancer variant interpretation and prioritization**

Supplemental materials

- Supplemental Method 1: Subclone structure simulation process.
- Supplemental Result 1: Comparison of performance among TrAp, PhyloSub, and SubcloneSeeker, and example of SubcloneSeeker utilizing CNV data based on microarray.
- Supplemental Figure 1: Subclone structure reconstruction results with different packages, based on SNP clusters of TCGA-13-0913.
- Supplemental Figure 2: Subclone structure reconstruction using microarray based copy number variation data in TCGA-13-0913.
- Supplemental Figure 3: Example of subclone analysis with SNP6 B-Allele Frequency probe intensity data.
- Supplemental Figure 4: Complete set of mutation co-localization prediction performance on simulated data.
- Supplemental Figure 5: Reported and analysis results on patient SU070 HSC sample in Jan *et al*.
- Supplemental Table 1: Summary of the re-analysis results of AML patient samples reported in Ding *et al*.
- Supplemental Table 2: Somatic variations used in the re-analysis of the HSC targeted deep sequencing dataset in Jan *et al*.
- Supplemental Table 3: Mutation co-localization frequency matrix for patient SU048 HSC targeted deep sequencing data from Jan *et al*.

**Supplemental Method 1: Subclone structure simulation process.**

In order to understand the behavior of our subclone reconstruction algorithm, we designed a tumor subclone structure simulator. The simulator initialize in a state that it only contains one subclone with no somatic event. This ‘null’ subclone logically represents the normal tissue before tumor expansion, and mathematically represents the normal tissue contamination usually found in tumor sample. We also assign a ‘viability’ value of 100 to this null subclone. The viability value represent the ability for a certain subclone to grow, and will ultimately determine the subclone frequency (SF) of each subclone. The simulator will now repeat the following steps exactly n times to simulate one subclone structure with n subclones.

1. From the existing subclones, a ‘parent’ subclone will be selected randomly by rolling a roulette wheel. The proportion of each subclone on the roulette wheel is determined by the viability value of the subclone.
2. A new subclone is created, with one additional mutation, and attached as a children node to the parent subclone. The mutation is only symbolic, so that allele frequency can be calculated at the end.
3. The viability value of the new subclone is determined by randomly sampling from a uniform distribution with a range of (0.5 * Parent’s Viability, 2 * Parent’s Viability), signifying that a mutation can be beneficial, detrimental, or neutral to the growth advantage.

The process is not meant to accurately model the actual tumor microevolution, but to create a large number of subclone structures with varying topology and cell prevalence. After the structure is created, each subclone is assigned a SF proportional to its viability value:

$${SF}_{i}=\frac{{Viability}_{i}}{\sum Viability}$$

The cell prevalence value for each of the introduced mutations will be calculated, which will serve as the input to the subclone reconstruction algorithm.

$${CP}_{j}=\sum_{i} {SF}_{i}\cdot B_{i}\text{; }B_{i}=\left\{ \begin{matrix} 1,\text{ if subclone }i\text{ contains mutation} j \\ 0, \text{otherwise} \end{matrix} \right.$$

The output of the simulation procedure will be a subclone structure, along with the CP value of all the mutations. The CP values will be used as input to the subclone reconstruction algorithm, and the subclone structure will be used to check if, among the results produced by the reconstruction, the correct structure has been found.

**Supplemental Result 1: Comparison of performance among TrAp, PhyloSub, and SubcloneSeeker,** **and example of SubcloneSeeker utilizing CNV data based on microarray.**

**Subclone reconstruction by TrAp [3] and Phylosub [4], using raw 454 sequencing read counts for each SNVs.** We first attempted to perform subclone reconstruction using the raw read counts of 21 validated somatic SNVs with 738x median and 1018x mean coverage, as this is the format these packages are designed to take as their input. However, TrAp (v0.3) issued an OutOfMemory error with 4G memory allocated to the JVM, and PhyloSub (commit 540fdfb003, as of 17 June 2014) produced a partial order plot that made little sense due to the high number of nodes and edges. See Additional file 3 for the actual dataset used for this test.

**Subclone reconstruction by SubcloneSeeker, using SNV clusters.** We clustered the same 21 SNPs on the primary / relapse allele frequency space, and identified four clusters (Supplemental Figure 1). SubcloneSeeker produced two structures with the primary clusters and one solution with the relapse clusters. One of the primary structures was trimmed away during the primary / relapse tree merging, resulting in a unique subclone structure for this patient.

Supplemental Figure 1, Subclone structure reconstruction results with different packages, based on SNP clusters of TCGA-13-0913. Left: The clusters, as well as their centroid allele frequency values Right: The primary, relapse, and merged primary / relapse pair structures identified by SubcloneSeeker.

**SubcloneSeeker’s unique ability to perform structure reconstruction on additional data types.** We obtained CNV segments from TCGA-13-0913 microarray level 2 probe intensity data (See Additional file 4 for the raw segmental data), and clustered them in primary / relapse CP space. The reconstruction result (Supplemental Figure 2) suggests the same conclusion as presented in the main text (Figure 6A, Supplemental Figure 1), although the exact structure for the primary tumor sample differs. This is because that, although these two datasets were from the same patient, the DNA samples are different preparations, resulting in different sampling on the underlying tumor cell population, and consequently would not necessarily correspond to the same subclone structure / fraction distribution, or that each could be providing a partial view on the overall subclone structure.

Supplemental Figure 2, Subclone structure reconstruction using microarray based Copy Number Variation data in TCGA-13-0913. (A) Probe Intensity of both the primary (TCGA-13-0913-01A) and relapse (TCGA-13-0913-02A) tumor sample. (B) CNV segments clustered on the primary / relapse cell prevalence space. (C) Subclone structure and relapse pattern from the identified clusters.


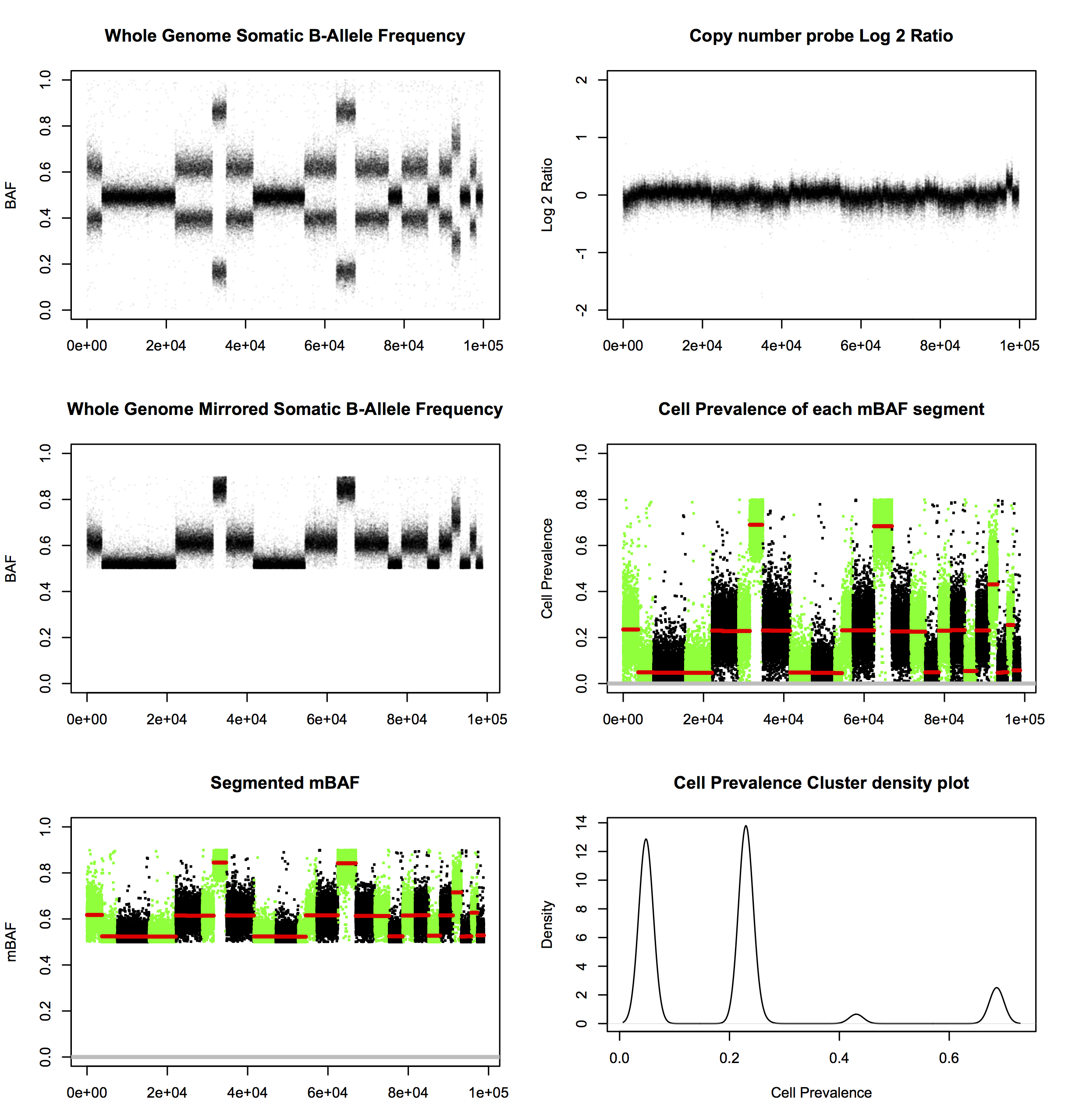


G

A

B

C

D

E

F

Supplemental Figure 3, Example of subclone analysis with SNP6 B-Allele Frequency probe intensity data. (A) The B-Allele frequency (BAF) data in JPII-32 tumor sample is filtered to only retain those that are heterozygous in the JPII-32 normal sample. (B) The mirrored BAF (mBAF) data is acquired by mapping all BAF data points smaller than 0.5 (denoted as x) to 1-x. (C) mBAF is then subjected to circular binary segmentation so that continuous segments of LOH can be identified. (D) The copy number probe Log 2 Ratio track of the SNP 6 array is shown to illustrate that there is no observable copy number alteration that is correlating with the observed LOH pattern, indicating that the multi-level LOH is a result of multi-clonality. (E) The segmented mBAF values are converted to cell pravelence value (CP). CP represents, for any given LOH event, what is the fraction of cells that are harboring the event, out of the entire cell population measured. (F) CP value clusters. (G) Biologically meaningful subclone structures that are consistent with the CP values. (A) – (E) was originally published in Nature [2]

Supplemental Figure 4. Complete set of mutation co-localization prediction performance on simulated data. C.SI - Sensitivity for co-localizing cells; C.PPV - Positive predictive value for co-localizing cells; NC.SI - Sensitivity for not co-localizing cells; NC.PPV - Positive predictive value for not co-localizing cells; SI - Combined sensitivity; PPV - Combined positive predictive value; AMB - Ambiguous cell fraction.

SU070 (Figure 10) HSC targeted deep sequencing data resulted in a unique solution (Figure 10C), because of the relatively high AF of the profiled mutations. This unique solution precisely supports the linear mutation acquisition model reported in Jan *et al.* (Figure 10A and B). In the colony assay, two colonies were identified to have TET2-Y1649STOP, but not TET2-T1884A, whereas in our result, these two mutations first appeared in the same subclone. Moreover, the AF data from bulk HSC deep sequencing suggests that TET2-T1884A (AF=48.10%) came before TET2-Y1649STOP (AF=47.87%) with only a very small difference in AF. This discrepancy is likely caused by AF inaccuracies from experimental error. Overall, our result successfully remodeled the linear mutation acquisition structure, and confirmed the conclusion that all these mutations in tandem were required for the AML tumorigenesis.

Supplemental Figure 5. Reported and analysis results on patient SU070 HSC sample in Jan *et al.* [1] (A) Colony assay results reported in Jan *et al.* (B) Evolution model reported in Jan *et al*. based on the colony assay results. (C) The unique evolution tree constructed from the deep sequencing results on heterogeneous HSC sample.

| Patient no. | Solutions based on primary sample (n) | Solutions based on relapse sample (n) | Compatible primary / relapse pairs (n) | Whether the results are in agreement with the model presented in the original paper |
| --- | --- | --- | --- | --- |
| 933124 | 6 | 1 | 1 | Yes |
| 758168 | 1 | 2 | 2 | No |
| 400220 | 1 | 1 | 1 | Yes |
| 426980 | 1 | 1 | 1 | Yes |
| 452198 | 1 | 1 | 1 | Yes |
| 573988 | 1 | 1 | 1 | Yes |
| 804168 | 1 | 1 | 1 | Yes |
| 869586 | 2 | 1 | 1 | Yes |

Supplemental Table 1. Summary of the re-analysis results of AML patient samples reported in Ding *et al.* [5].

| Patient | Mutation | Variant allele read count | Reference allele read count | Variant AF |
| --- | --- | --- | --- | --- |
| SU008 | SKP2 | 45,937 | 624,754 | 0.068492048 |
| SU008 | ELP2 | 1,915 | 504,335 | 0.003782716 |
| SU008 | PDZD3 | 161 | 100,433 | 0.001600493 |
| SU008 | CNDP1 | 2,238 | 475,621 | 0.00468339 |
| SU030 | KCTD4 | 116,061 | 2,090,267 | 0.052603693 |
| SU030 | SLC12A1 | 7,754 | 1,163,598 | 0.006619701 |
| SU048 | ACSM1 | 16,819 | 110,087 | 0.132531165 |
| SU048 | NPM1 | 30 | 11,079 | 0.002700513 |
| SU048 | OLFM2 | 13,717 | 108,695 | 0.112056008 |
| SU048 | PYHIN1 | 16 | 12,952 | 0.001233806 |
| SU048 | SMC1A | 181,167 | 477,095 | 0.275220201 |
| SU048 | TET2-D1384V | 1,797 | 15,854 | 0.101807263 |
| SU048 | TET2-E1357STOP | 7,416 | 12,117 | 0.379665182 |
| SU048 | ZMYM3 | 18,518 | 288,810 | 0.060254842 |
| SU070 | TET2-Y1649STOP | 7,732 | 8,419 | 0.478731967 |
| SU070 | CXOFF36 | 3,503 | 4,537 | 0.435696517 |
| SU070 | CACNA1H | 12,083 | 12,775 | 0.48608094 |
| SU070 | TET2-T1884A | 4,218 | 4,552 | 0.480957811 |
| SU070 | CXOFF66 | 3,678 | 4,466 | 0.451620825 |
| SU070 | SCN4B | 5,086 | 11,273 | 0.310899199 |
| SU070 | NCRNA00200 | 9,199 | 16,212 | 0.362008579 |
| SU070 | GABARAPL1 | 1,648 | 3,344 | 0.330128205 |
| SU070 | DOCK9 | 3,382 | 5,285 | 0.390215761 |
| SU070 | CTCF | 10,529 | 19,561 | 0.349916916 |
| SU070 | PXDN | 78 | 4,712 | 0.016283925 |
| SU070 | TMEM20 | 157 | 14,986 | 0.010367827 |
| SU070 | TMEM8B | 69 | 7,791 | 0.008778626 |

Supplemental Table 2. Somatic variations used in the re-analysis of the HSC targeted deep sequencing dataset in Jan *et al.* [1].

| **Mutation co-localization frequency matrix** | | | | | |
| --- | --- | --- | --- | --- | --- |
|  | TET2-E1357STOP | SMC1A | ACSM1 | OLFM2 | TET2-D1384V |
| SMC1A | 1 |  |  |  |  |
| ACSM1 | 1 | 1 |  |  |  |
| OLFM2 | 0.67 | 0.67 | 0.33 |  |  |
| TET2-D1384V | 0.75 | 0.5 | 0.25 | 0.25 |  |
| ZMYM3 | 0.75 | 0.5 | 0.25 | 0.25 | 0.25 |

Supplemental Table 3. Mutation co-localization frequency matrix for patient SU048 HSC targeted deep sequencing data from Jan *et al.* [1]. Mutations are sorted in descending order by AF.

References

1. Jan M, Snyder TM, Corces-Zimmerman MR, Vyas P, Weissman IL, Quake SR, Majeti R: **Clonal evolution of preleukemic hematopoietic stem cells precedes human acute myeloid leukemia.** *Sci Transl Med* 2012, **4:**149ra118.

2. Wang L, Yamaguchi S, Burstein MD, Terashima K, Chang K, Ng HK, Nakamura H, He Z, Doddapaneni H, Lewis L, Wang M, Suzuki T, Nishikawa R, Natsume A, Terasaka S, Dauser R, Whitehead W, Adekunle A, Sun J, Qiao Y, Marth G, Muzny DM, Gibbs RA, Leal SM, Wheeler DA, Lau CC: **Novel somatic and germline mutations in intracranial germ cell tumours.** *Nature* 2014, **511:**241-245.

3. Strino F, Parisi F, Micsinai M, Kluger Y: **TrAp: a tree approach for fingerprinting subclonal tumor composition.** *Nucleic Acids Res* 2013, **41:**e165.

4. Jiao W, Vembu S, Deshwar AG, Stein L, Morris Q: **Inferring clonal evolution of tumors from single nucleotide somatic mutations.** *BMC Bioinformatics* 2014, **15:**35.

5. Ding L, Ley TJ, Larson DE, Miller CA, Koboldt DC, Welch JS, Ritchey JK, Young MA, Lamprecht T, McLellan MD, McMichael JF, Wallis JW, Lu C, Shen D, Harris CC, Dooling DJ, Fulton RS, Fulton LL, Chen K, Schmidt H, Kalicki-Veizer J, Magrini VJ, Cook L, McGrath SD, Vickery TL, Wendl MC, Heath S, Watson MA, Link DC, Tomasson MH, et al: **Clonal evolution in relapsed acute myeloid leukaemia revealed by whole-genome sequencing.** *Nature* 2012, **481:**506-510.
